# Bacterial Communities of the Canola Rhizosphere: Network Analysis Reveals a Core Bacterium Shaping Microbial Interactions

**DOI:** 10.3389/fmicb.2020.01587

**Published:** 2020-07-10

**Authors:** Jean-Baptiste Floc’h, Chantal Hamel, Newton Lupwayi, K. Neil Harker, Mohamed Hijri, Marc St-Arnaud

**Affiliations:** ^1^Institut de Recherche en Biologie Végétale, Université de Montréal and Jardin Botanique de Montréal, Montreal, QC, Canada; ^2^Quebec Research and Development Centre, Agriculture and Agri-Food Canada, Quebec City, QC, Canada; ^3^Lethbridge Research and Development Centre, Agriculture and Agri-Food Canada, Lethbridge, AB, Canada; ^4^Lacombe Research and Development Centre, Agriculture and Agri-Food Canada, Lacombe, AB, Canada; ^5^AgroBiosciences, Mohammed VI Polytechnic University, Ben Guerir, Morocco

**Keywords:** bacteria, archaea, microbial ecology, agroecosystem, crop rotations, *Brassica napus*

## Abstract

The rhizosphere hosts a complex web of prokaryotes interacting with one another that may modulate crucial functions related to plant growth and health. Identifying the key factors structuring the prokaryotic community of the plant rhizosphere is a necessary step toward the enhancement of plant production and crop yield with beneficial associative microorganisms. We used a long-term field experiment conducted at three locations in the Canadian prairies to verify that: (1) the level of cropping system diversity influences the α- and β-diversity of the prokaryotic community of canola (*Brassica napus*) rhizosphere; (2) the canola rhizosphere community has a stable prokaryotic core; and (3) some highly connected taxa of this community fit the description of hub-taxa. We sampled the rhizosphere of canola grown in monoculture, in a 2-phase rotation (canola-wheat), in a 3-phase rotation (pea-barley-canola), and in a highly diversified 6-phase rotation, five and eight years after cropping system establishment. We detected only one core bacterial Amplicon Sequence Variant (ASV) in the prokaryotic component of the microbiota of canola rhizosphere, a hub taxon identified as cf. *Pseudarthrobacter* sp. This ASV was also the only hub taxon found in the networks of interactions present in both years and at all three sites. We highlight a cohort of bacteria and archaea that were always connected with the core taxon in the network analyses.

## Introduction

A plant in its natural environment coexists with myriads of archaea, bacteria, fungi, as well as with other unicellular eukaryotic microorganisms that constitute its microbiota. The rhizosphere is a hotspot of microbial interactions between species that have various ecological functions. These microbial communities are particularly important for plant health as they influence its development and its productivity (Barriuso et al., 2008; Bulgarelli et al., 2013; Bakker et al., 2014). Throughout their life, plant roots exude compounds creating the rhizosphere environment (Bais et al., 2006). Spatial and temporal variation in rhizodeposition allows plants to shape their rhizosphere microbial communities to their benefit (Tkacz et al., 2015; Pii et al., 2016; Eisenhauer et al., 2017).

Plant rhizosphere can host mutualistic microbes such as mycorrhiza or plant growth promoting bacteria (PGPB) that facilitate nutrient uptake, mitigate abiotic stress, and prevent root infection by pathogens (Barriuso et al., 2008; Farina et al., 2012; Fincheira and Quiroz, 2018). Plant-microbe and microbe-microbe interactions are diverse. Plants live in symbiotic and commensal relationships with numerous organisms, but they must also face pathogenic attacks (Hajishengallis et al., 2012). Rhizosphere organisms may influence each other, thus forming a complex web of interactions. For example, we know that mycorrhizal fungi have their own bacterial microbiota (Bianciotto et al., 2003; Iffis et al., 2014, 2017). These bacteria can be endophytic or form biofilm at the surface of the hyphae and can facilitate symbiosis formation in plants (Fitter and Garbaye, 1994; Iffis et al., 2014; Taktek et al., 2017).

Since the last decade, new generation sequencing (NGS) improved our access to microbial genetic information leading to significant advances in microbial ecology. This technological improvement lead to new ways of analyzing plant microbial communities (Duffy et al., 2007; Bulgarelli et al., 2013; Mendes et al., 2015). Now, we can identify with confidence the factors shaping the microbial communities of the rhizosphere (Kuramae et al., 2011; Agler et al., 2016). The microbiome of the rhizosphere is extremely large and diverse. To summarize this complexity, we can divide it into pools of microbes based on their functions or occurrence (Ridout and Newcombe, 2016). In a given community, microbial taxa are likely to be favored by their host plant throughout its existence (Rout, 2014). These taxa are expected to be always part of the plant microbiota at a defined time *t*, regardless of environmental conditions. According to Vandenkoornhuyse et al. (2015), the taxa always present in association with the plant forms the core microbiome and have preferential interaction with their host. The definition of a pool of microorganisms always present at t time in the plant microbiota is appropriate for most ecological studies concerning the plant microbiota as they mostly rely on a single sampling time. However, it was necessary to consider temporal variation in our definition of the core microbiota, and this is what we did in this study.

The interactions between microbes in the plant rhizosphere remains largely obscure. Next Generation Sequencing techniques can provide information on the abundance of the taxa interacting in a microbiome, but cannot reveal the biochemistry of interacting microbes in the ecosystem. That is why computational approaches aiming at identifying the nature of the links between the variations in the abundance of microbial taxa were developed as a complement to NGS (Ings et al., 2009; Deng et al., 2012; van der Heijden and Hartmann, 2016). Network analysis allows us to identify microbial taxa that are functionally linked to others within the microbiome. Highly connected microorganisms may have a greater impact on plants and ecosystem functioning than others, because they theoretically interact with many partners and antagonists; these highly interacting species are named hub taxa (Agler et al., 2016). Interactions occurring in microbial communities are known to be complex and difficult to retrieve with usual statistical methods (Kurtz et al., 2015). However, the information provided by NGS can be processed through network analysis to identify cohorts represented by hub taxa. Simplifying the study of complex microbiome, Taktek et al. (2017) showed taxa that recruit organisms beneficial to the host plant, but hub taxa could also be pathogens. Some hub taxa in the human microbiome can articulate infection by consortia of pathogens (Hajishengallis et al., 2012). As pathogens can affect the plant microbiome, pathogenic hub taxa may occur in the rhizosphere. The hub taxa are a useful concept and help to understand the ecology of the root and rhizosphere ecosystems, which could lead to the development of applications in crop plant root systems.

Canola was shown to possess a specific bacterial component of the core microbiota conserved across the Canadian prairie (Lay et al., 2018). Floc’h et al. (2020) reported the temporal stability of the fungal component of the core microbiota in canola rhizosphere, despite considerable changes in the plant rhizosphere microbiota across years. In the present study, we aimed to test if the bacterial component of the canola microbiota has a similar pattern of temporal variation. We investigated the temporal stability of the bacterial component of the core canola rhizosphere microbiota in order to ascertain whether a persistent bacterial component exists. Another aim was to determine if the canola rhizosphere harbors bacterial hub taxa, and to visualize the variation between years in the structure of interactions among the bacteria living in the canola rhizosphere microbiota. We sought to identify a universal bacterial component of the core microbiota in the rhizosphere of a plant species, specifically canola grown over the years under a range of climatic conditions and biological environments. We thus used a gradient of crop diversification levels to create variation in the biological environment of rhizosphere soil and examine over two years what in the bacterial component of the canola microbiota is invariable: the core microbiota. Canola is a crop of economical importance for Canada. It is also a good model plant to study the rhizosphere microbiome as canola produce antimicrobial isocyanates (Zheng et al., 2014) leading to simpler microbial communities in its rhizosphere (Rumberger and Marschner, 2003).

## Materials and Methods

Three sites located in three pedoclimatic zones of the canola-producing area of western Canada were used. Two sites were in Alberta, specifically in Lacombe (lat. 52.5°N, long. 113.7°W) and Lethbridge (lat. 49.7°N, long. 112.8°W), and the third site was in Scott, Saskatchewan (lat. 52.4°N, long 108.8°W). The soil in Lethbridge is a Brown Chernozem with a silty loam texture, while the Dark Brown Chernozems have a loamy texture at the Scott site and a clay loam texture at Lacombe.

Plots of a larger long-term crop rotation experiment initiated in 2008 were used for this study. Site description, experimental design and sampling methods are described in details in Floc’h et al. (2020). This study had a complete randomized block design replicated at three geographic sites, each with 4 blocks and 4 crop rotation treatments, and we collected samples from the Roundup Ready (RR) canola phase of the crop rotations on 2 years, in 2013 and 2016. The four treatments were four levels of cropping system diversification: (1) monoculture of RR canola, (2) wheat-RR canola, (3) pea-barley-RR canola, and (4) lentil-wheat-Liberty Link canola-pea-barley-RR canola (Table 1). Crops were grown according to best management practices. Information on crop management is described in Harker et al. (2015).

**TABLE 1:** Selected treatments from a long-term experiment established in 2008 at three different sites in the Canadian prairies (Harker et al., 2015).

	Cropping systems	
Diversification level	2008–2013	2008–2016
Monoculture	RR-RR-RR-RR-RR-RR^1^	RR-RR-RR-RR-RR-RR-RR-RR-RR
Low	W-RR-W-RR-W-RR	RR-W-RR-W-RR-W-RR-W-RR
Medium	P-B-RR-P-B-RR	P-B-RR-P-B-RR-P-B-RR
High	Len-W-LL^2^-P-B-RR	Len-W-LL-P-B-Len-W-P-RR

*The rotation phases examined in this study in 2013 and 2016 are underlined. ^1^RR, canola 71-45, a Roundup Ready cultivar resistant to glyphosate. ^2^LL, canola InVigor 5440 Liberty Link, cultivar resistant to glufosinate.*

Rhizosphere samples were collected during the fourth week of July in 2013 and 2016, which corresponds to the end of canola flowering period. Three to four plants randomly selected within each plot were uprooted with a shovel. The shoots were removed and roots were placed in plastic bags and brought to the laboratory on ice in a cooler. About 5 g of rhizosphere soil per plot was collected by gently brushing the roots. The samples were kept at 4°C before being shipped on ice to Lethbridge, Alberta, where they were preserved at -80°C until DNA extraction.

### DNA Extraction and Amplification

DNA extraction was conducted as described in Floc’h et al. (2020). We constructed amplicon libraries for bacterial 16S rRNA gene sequences by using target-specific PCR primers attached to Illumina overhang sequences for NextEra library preparation. The primer pairs were GTGCCAGCMGCCGCGGTAA (515F-Illu) and GGACTACHVGGGTWTCTAAT (806R-Illu). This primer set was selected because it is used by the Earth Microbiome Project.^1^ Two PCR reactions were performed to prepare the amplicon library. In the first PCR reaction, the V4 hypervariable region of prokaryotic 16S RNA genes was amplified using primers previously described (515F and 806R). The PCR reaction was performed in a 25-μL reaction mixture containing 1 μL of template DNA, 1 × PCR-buffer (Qiagen, Germantown, MD, United States), 1.8 mM MgCl_2_, 1.25 μL of 5% dimethylsulfoxide (DMSO), 0.2 mM dNTP, 0.5 U Taq DNA polymerase (Roche, Branford, CT, United States), and 0.6 μM of each primer. The 5′ ends of the forward and reverse primers were tagged with CS1 (ACACTGACGACATGGTTCTACA) and CS2 (TACGGTAGCAGAGACTTGGTCT), respectively, which were used as anchors for the PCR reaction. The conditions to amplify the prokaryotic 16S rRNA fragments consisted of an initial denaturation at 94°C for 2 min, 33 cycles of denaturation at 94°C for 30 s, annealing at 58°C for 30 s and elongation at 72°C for 30 s, followed by a final elongation at 72°C for 7 min.

The second PCR reaction was used to add barcodes to each sample and the Illumina sequencing adapters. This PCR reaction was performed in a 20-μl reaction mixture, containing 1 × PCR-buffer (Qiagen, Germantown, MD, United States), 1.8 mM MgCl_2_, 1 μl of 5% DMSO, 0.2 mM dNTP, 0.5 U Taq DNA polymerase (Roche, Branford, CT, United States), 2 μM of NextEra XT index primers (Illumina Inc., San Diego, CA, United States), and 1 μL of 1/150 dilution of the first PCR products. The PCR conditions were as follows: initial denaturation at 95°C for 10 min, 15 cycles of denaturation at 95°C for 15 s, annealing at 60°C for 30 s, and elongation at 72°C for 1 min followed by a final elongation at 72°C for 3 min. After the second amplification, PCR products were quantified using Quant-iT^TM^ PicoGreen^®^ dsDNA Assay Kit (Life Technologies, Canada) and the Kapa Illumina GA with Revised Primers-SYBR Fast Universal kit (D-Mark, Canada). The amplicon library was purified using calibrated AMPure XP beads (Agencourt, United States), and the average size and quantity of each library were assessed on the LabChip GX (Perkin Elmer, United States) instrument. The library was then sequenced on Illumina MiSeq using the paired-end 250 protocol at Génome Québec Innovation Centre at McGill University (Montreal, Canada).

### ASV Determination and Bioinformatic Pipeline

The bioinformatic pipeline used for the processing of our 16S rRNA gene sequences from 2013 and 2016 was DADA2 v1.8 (Callahan et al., 2016). We first used Cutadapt 1.13 to remove the primer part of the 16S rRNA gene sequences. Then, we excluded the sequences with less than 200 bp as the base quality of the sequences tended to diminish below that threshold in our data with the command “filterAndTrim” with a “maxEE” score of 2, “trunQ” score of 2 and “minLen” argument set to 50. Then, we calculated the error rate using the machine learning algorithm implemented in DADA2 with the command “learnErrors.” As the error rate was satisfying according to developer’s recommendations, we merged the forward and reverse sequences using the command “mergePairs.” Afterward, the Amplicon Sequence Variant (ASV) table was calculated and the chimeras eliminated using the command “makeSequenceTable,” resulting in a sequence length ranging from 250 to 253 nucleotides. ASVs were then identified using the naïve Bayesian classifier method on the databases SILVA and RDP, and the identity of ASVs of interest was verified manually using BLAST on the NCBI nt database. With the taxonomic resolution of the 16S RNA gene, it is generally not possible to identify a bacterium at the species level. Thus, the identifications at species level presented here must be consider with caution despite they perfectly match (100% similarity and coverage) the reference sequences of NCBI.

The MiSeq sequencing data generated as part of this work are publicly available on Zenodo.^2^

### Data Processing and Statistical Analyses

We first wanted to assess the variation occurring in canola rhizosphere caused by the crop diversification systems. The dataset was standardized by randomly subsampling the read data from each sample to the lowest number of reads (13 241) encountered for a sample, using the function “rrarefy” of the vegan package v.2.4.6 in R v. 3.4.3, before calculating Chao1 (Chao, 1984), Shannon and Simpson’s *α*-diversity indices using the same package.

The significance of crop diversification effect on *α*-diversity indices was tested by analysis of variance (ANOVA) one year at a time, combining sites and blocks in one random effect with 12 blocks (four blocks per each of the three sites), and comparisons between treatment means were made with Tukey’s *post-hoc* tests using the R package agricolae v1.3.1 (Mendiburu, 2015). The effect of crop diversification on bacterial community structure was assessed by permutational multivariate analysis of variance (PERMANOVA) (Anderson, 2001), considering 12 blocks (four blocks per each of the three sites), using the function “adonis” of the vegan package v 2.4.6 (Oksanen et al., 2019) in R v3.4.3, and the entire (non-subsampled) set of relative abundance data. The blocked multi-response permutation procedure (BMRPP) was used for pairwise comparison of community structure under the different crop diversification treatments. using Šidák correction for pairwise comparison in the R package “RVAideMemoire” v0.9 (Hervé, 2015).

After determining the impact of crop diversification on canola rhizosphere, we aimed at identifying its universal bacterial component of the core microbiota and hub taxa. We defined the core microbiota as the set of organisms that are present in the microbiota at all sites and plots at t and t+1. To assess the interactions among bacterial taxa in the microbiota, we created a co-occurrence network using the package SPIEC-EASI v 1.0.6 in R 3.4.3 (Kurtz et al., 2015). The analysis was conducted over all bacterial rhizosphere communities of each year. The input data consisted in the matrix of the raw abundance of ASVs of one year of sampling. We first filtered the dataset to remove the ASVs with a frequency less than 20%. The SPIEC-EASI run was done with the algorithm “mb” with the lambda min ratio set at 10^–2^ and 50 repetitions. We then imported the networks in Cytoscape 3.7.1 (Smoot et al., 2011) for plotting and used the “organic” layout to draw the network. Edges where defined as co-occurrences or mutual exclusion regarding the positives or negatives values of inverse covariance linking the nodes. Betweenness centrality, defined as the fraction of the shortest path between all other nodes in the network containing the given node, and degree score, highlight central nodes and provide information about network architecture. A score of betweenness centrality and degree of connectivity greater than the score of 95% of the network taxa could suggest participation in multipartite interactions in the community and allow us to flag the highly connected taxa as hub-taxa. Hub-taxa were defined as the nodes possessing a score of betweenness centrality > 0.40 and a degree score > 10.

Spearman’s correlations between abundance of hub-taxa and of their cohorts with canola yield were computed on R 3.4.3.

## Results

### Taxonomic Affiliation of the Bacterial Component of the Canola Rhizosphere Microbiota

**FIGURE 1:**
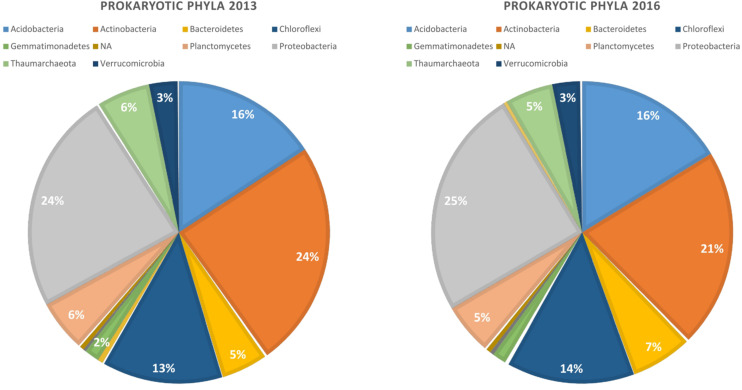
Relative abundance of dominant bacterial phyla in the rhizosphere of canola in 2013 and 2016.

Our bioinformatic pipeline retrieved 2 175 992 reads from the 96 samples, that were assigned to 10 385 ASVs. Read number per sample ranged from 10 938 to 60 896. The ASVs belong mostly to four bacterial phyla that did not vary substantially in abundance in the two years of study: *Proteobacteria* (25%), *Actinobacteria* (22.5%), *Acidobacteria* (16%), and *Chloroflexi* (13%) (Figure 1). Rarefaction curves indicated that read abundances were close to saturation for all the samples (Supplementary Figure S2).

### Effect of Treatments on Communities

Crop diversification had no significant influence on *α*-diversity indices (Table 2) or on the structure of bacterial communities of canola rhizosphere in 2013 (Table 3). On the other hand, crop diversification significantly affected the community structure of canola rhizosphere in 2016 by PERMANOVA (*P* = 0.047), where the rhizosphere communities of canola in monoculture and in the highly diversified system were structurally different according to the BMRPP test (Table 3). PCoA analyses showed a clear segregation of prokaryotic communities by site, but did not show clear patterns between diversification levels either in 2013 and 2016 (Supplementary Figures S3, S4). Since the prokaryotic communities segregated per site, additional PERMANOVA were conducted to assess whether, within each site, the crop diversification level has an effect on the microbiota structure. Results did not show any differential effect of crop diversification per site and year (Table 3). Indicator species analysis revealed ASVs significantly associated with crop diversifications. In 2013, the highest level of crop diversification had the highest number of indicator species (15), whereas monoculture had nine and the diversification treatment with wheat and canola had only one (Table 4). No indicator species was found in association with the medium crop diversification level in 2013. In 2016, monoculture showed the highest number of indicator species with 26 ASVs, the low crop diversification had four and the medium diversification had one. No indicator species were found in association with the highest level of crop diversification in 2016. ASV108 (cf. *Thermomicrobiales* sp.) was and indicator species of the monoculture in 2013 and 2016; it is also the only indicator species to be found in both years of sampling.

**TABLE 2:** Mean values of bacterial *α*-diversity indices in the rhizosphere of canola under different crop diversification levels, in 2013 and 2016.

	2013				2016			
Index^1^	Monoculture^2^	Low	Medium	High	Monoculture	Low	Medium	High
Shannon	5,256	5,297	5,318	5,219	5,397	5,321	5,183	5,227
Simpson	0,990	0,989	0,990	0,988	0,990	0,989	0,985	0,986
Chao1	347,961	385,078	394,167	365,999	441,829	429,286	396,087	428,412
Richness	346,467	382,942	396,768	364,275	436,833	423,442	390,867	420,333

*^1^No significant differences in diversity index values were detected between the crop rotations by Tukey test α = 0.05. ^2^Monoculture, canola monoculture; Low, wheat-canola rotation; Medium, pea-barley-canola rotation; High, lentil-wheat-LL-pea-barley-RR.*

**TABLE 3:** Effects of crop diversification on the structure of the bacterial community in the canola rhizosphere, in 2013 and 2016, according to PERMANOVA (*α* = 0.05, *n* = 12), and significant differences between the structure of bacterial communities per crop diversification level according to Blocked Multi-Response Permutation Procedures (BMRPP) with Šidák correction for two-way comparisons (*α* = 0.035, *n* = 12).

	PERMANOVA			
	2013		2016	
Source	DF^1^	*P*-value	DF	*P*-value
**Overall model**				
Crop diversification	3	0,202	3	0,047*
Residuals	44		44	
**Lacombe**				
Crop diversification	3	0,88	3	0,262
Residuals	12		12	
**Lethbridge**				
Crop diversification	3	0,131	3	0,292
Residuals	12		12	
**Scott**				
Crop diversification	3	0,319	3	0,479
Residuals	12		12	
**MRPP**				
Monoculture^2^	a^3^		a	
Low	A		ab	
Medium	A		ab	
High	A		b	

*^1^DF : Degree of Freedom. ^2^Monoculture, canola monoculture; Low, wheat-canola rotation; Medium, pea-barley-canola rotation; High, lentil-wheat-LL-pea-barley-RR. ^3^Within each column, crop rotations associated with the same letters are not significantly different.*

**TABLE 4:** Indicator species analysis of the prokaryotic ASV residing in the rhizosphere of canola in response to cropping diversification treatment in 2013 and 2016.

	2013			2016		
Crop diversification^1^	Indicator species ASV	Closest identity	*P* value	Indicator species ASV	Closest identity	*P* value
**Monoculture**	ASV315	*Thermomicrobiales*	0,003**	ASV399	*Acidobacteria* sp.	0,002**
	ASV833	*Paracoccus* sp.	0,001**	ASV202	*Thermomicrobiales*	0,003**
	ASV380	*Chloroflexi* sp.	0,003**	ASV309	*Actinobacteria* sp.	0,002**
	ASV409	*Haliangium* sp.	0,010*	ASV576	*Thermomicrobiales*	0,007**
	ASV16	*Intrasporangiaceae*	0,026*	ASV276	*Chloroflexi* sp.	0,002**
	**ASV108^2^**	*Thermomicrobiales*	0,031*	ASV848	*Micromonosporaceae*	0,009**
	ASV280	*Chthoniobacter* sp.	0,042*	ASV119	*Rhizobiaceae*	0,013*
	ASV251	*Chthoniobacter* sp.	0,042*	ASV547	*Chloroflexi* sp.	0,003**
	ASV838	*Rhodanobacteraceae*	0,049*	ASV680	*Tepidisphaera* sp.	0,009**
				ASV334	*Rhizobiaceae*	0,015*
				ASV809	*Planctomycetes*	0,020*
				ASV315	*Thermomicrobiales*	0,022*
				ASV321	*Thermomicrobiales*	0,034*
				ASV460	*Chloroflexi* sp.	0,032*
				ASV60	*Gaiella* sp.	0,037*
				ASV142	*Chthoniobacter* sp.	0,030*
				ASV181	*Tepidisphaerales*	0,034*
				ASV629	*Solirubrobacter* sp.	0,025*
				ASV552	*Pseudonocardia* sp.	0,036*
				ASV137	*Rubinisphaeraceae*	0,045*
				ASV613	*Chloroflexi* sp.	0,043*
				**ASV108**	*Thermomicrobiales*	0,039*
				ASV463	*Parafilimonas* sp.	0,041*
				ASV227	*Acidobacteria*	0,045*
				ASV183	*Chitinophagaceae*	0,047*
				ASV1287	*Pirellula* sp.	0,047*
**Low**	ASV529	*Rubrobacter* sp.	0,015*	ASV501	*Pyrinomonadaceae*	0,001**
				ASV577	*Streptosporangium* sp.	0,008**
				ASV377	*Lysobacter* sp.	0,030*
				ASV697	*Frankiales*	0,036*
**Medium**				ASV1624	*Acidobacteria*	0,033*
**High**	ASV182	*Pseudomonas* sp.	0,002**			
	ASV214	*Gaiellales*	0,009**			
	ASV34	*Gaiella* sp.	0,012*			
	ASV283	*Haloactinopolyspora* sp.	0,014*			
	ASV498	*Rhizobiales*	0,014*			
	ASV624	*Bacteria*	0,017*			
	ASV93	*Nitrososphaeraceae*	0,016*			
	ASV751	*Acidobacteria*	0,003**			
	ASV59	*Holophagae* sp.	0,027*			
	ASV24	*Burkholderiaceae*	0,027*			
	ASV53	*Nitrososphaeraceae*	0,025*			
	ASV248	*Iamia* sp.	0,029*			
	ASV127	*Acidobacteria*	0,046*			
	ASV262	*Sphingomonas* sp.	0,040*			
	ASV302	*Acidobacteria*	0,048*			

Indicator values (IndVal) were tested for significance by Monte Carlo permutation tests (α = 0.05, 999 permutations). ^1^Monoculture, canola monoculture; Low, wheat-canola rotation; Medium, pea-barley-canola rotation; High, lentil-wheat-LL-pea-barley-RR. An empty row indicates the absence of indicator species. ^2^ASV in bold are indicator species found in 2013 and 2016 in the same crop diversification. Level of significance, *p < 0.05, **p < 0.01.

### Core Bacterial Component of the Canola Rhizosphere Microbiota

Only one bacterial ASV remained present across all the samples in every crop rotation and both years: ASV1. ASV1 was identified as cf. *Pseudarthrobacter* sp. according to SILVA and RDP databases and was the most abundant bacterial ASV in the canola rhizosphere in both years of the study. Its relative abundance ranged from 3.4% of the bacterial community in 2013 to 2.6% in 2016 and was not influenced by cropping system diversification.

### Network Analysis of the Bacterial Component of the Microbiota

**FIGURE 2:**
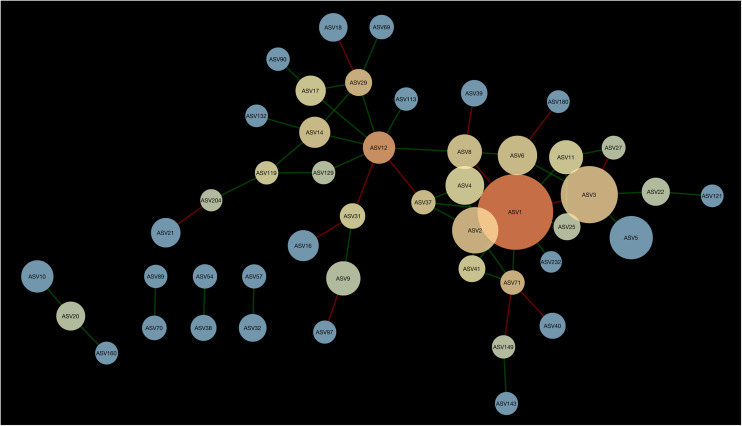
Network of interactions between bacteria forming the microbiome of canola rhizosphere in 2013. Dot size is proportional to the relative abundance of ASV, and shades indicate the degree of betweenness centrality: ASVs with warm colors are more connected with the other members of the network than the cold colored ones. Green edges indicate positive relationships and red edges, negative relationships.

**FIGURE 3:**
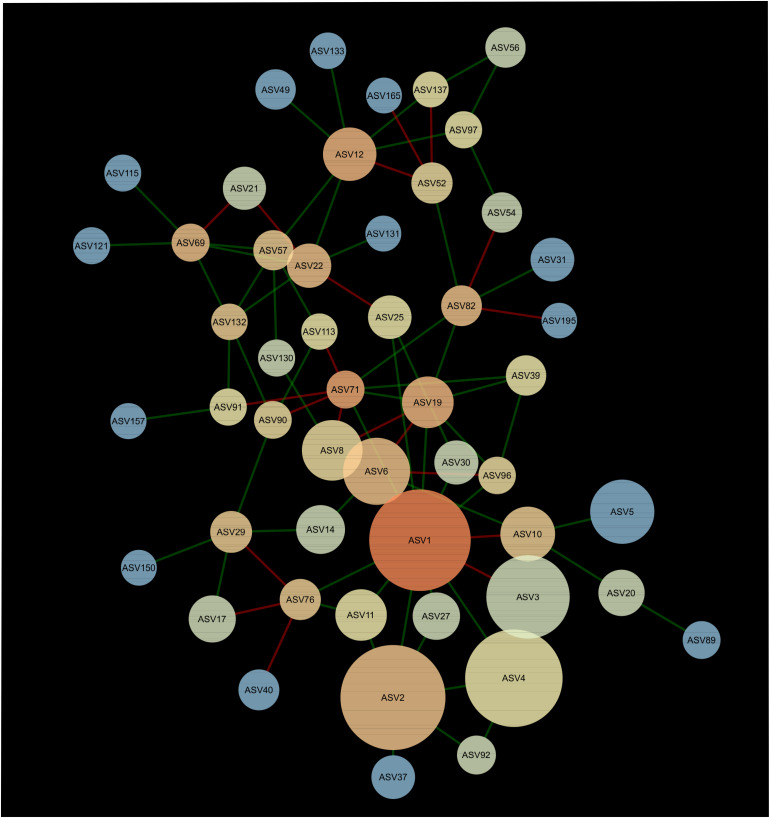
Network of interactions between the bacteria forming the microbiome of canola rhizosphere in 2016. Dot size is proportional to relative abundance of ASV, and shades indicate the degree of betweenness centrality: ASVs with warm colors are more connected with the other members of the network than the cold colored ones. Green edges indicate positive relationships and red edges, negative relationships.

A network composed of 47 ASVs and 56 edges was found in 2013 (Figure 2). This network was modular and included 13 mutual exclusions and 43 co-occurrences between bacterial taxa. A module was organized around ASV12 (cf. *Acidobacteria* sp.) which shared 5 co-occurrences and 2 mutual exclusions. Another module was organized around ASV1 (cf. *Pseudarthrobacter* sp.) which shared 9 co-occurrences and 3 mutual exclusions with other bacterial taxa. In 2016, the interaction network between bacteria was more complex than in 2013, with 51 ASVs and 83 edges (Figure 3). The network showed no modularity but was organized on ASV1 which shared 10 co-occurrences and 3 mutual exclusions with the other members of the network. Taxonomical affiliation of the ASVs of the networks in 2013 and 2016 can be found in Supplementary Tables S1, S2.

There was one ASV identified as hub-taxa in 2013 and in 2016, ASV1 (cf. *Pseudarthrobacter* sp.), that was also the only relatively abundant member of the bacterial component of the canola rhizosphere microbiota. In 2013, ASV1 had a score of betweenness centrality of 0.44 and a degree score of 11, and in 2016 a score of betweenness centrality of 0.44 and a degree score of 13. No other ASV of the networks had values of betweenness centrality and degree score above the threshold of 95% as it was the case for ASV1. We were able to identify a cohort of bacterial taxa that were connected with ASV1 in 2013 and in 2016 (Table 5). The types of interaction between ASV1 and its cohort members were consistent and stable through years. In particular, ASV1 was always positively linked with ASV2 (cf. *Yersinia* sp.), ASV4 (cf. *Stenotrophomonas* sp.), ASV11 (cf. *Stenotrophomonas* sp.), ASV25 (cf. *Candidatus Nitrosocosmicus* sp.) and ASV71 (cf. *Paenarthrobacter* sp.), and negatively linked with ASV3 (cf. *Nitrosophaeraceae* sp.) and ASV6 (cf. *Chloroflexi* KD4-96).

**TABLE 5:** Spearman’s correlation between the hub taxa ASV1 and its cohort members with canola yield (*N* = 48) in 2013 and 2016.

ASV^1^	Identity	Hub taxa^2^	2013		2016	
			% relative abundance	Spearman r	% relative abundance	Spearman r
ASV1	*Pseudarthrobacter* sp.	Y	3,430	ns	2,692	0,462*
ASV2	*Yersinia* sp.	N	1,599	ns	2,829	ns
ASV3	*Nitrosphaeraceae*	N	2,275	ns	1,981	0,286*
ASV4	*Stenotrophomonas* sp.	N	1,129	ns	2,533	ns
ASV6	*Chloroflexi KD4-96*	N	1,172	−0,400***^2^	1,341	−0,412***
ASV11	*Stenotrophomonas* sp.	N	0,831	ns	0,734	Ns
ASV25	*Candidatus Nitrosocosmicus* sp.	N	0,397	ns	0,422	Ns
ASV71	*Paenarthrobacter* sp.	N	0,234	ns	0,203	0,45***

^1^ASV: Amplicon Sequence Variant. ^2^Taxa with high connectivity in network analysis in 2013 and 2016 (see section “Materials and Methods” for details). Level of significance, *p < 0.05, **p < 0.01, and ***p < 0.001.

### Correlation Between ASV1 and Its Cohort Members, and Canola Yield

Spearman’s correlations were used to assess the relationship between ASV1 and its cohort members and canola yield in each years (Table 5). ASV1 and most of its cohort members were not related to canola yield in 2013, only ASV6 showed a moderate negative correlation (*R* = -0.40, *P* = 0.0149) with canola grain yield, according to Ratner (2009). However, in 2016, ASV1 was positively correlated with canola yield (*R* = 0.46, *P* = 0.001), as it was the case for ASV3 (*R* = 0.23, *P* = 0.05) and ASV71 (*R* = 0.45, *P* = 0.0012). ASV6 remained negatively correlated with canola yield (*R* = -0.41, *P* = 0.003).

## Discussion

We validated that a core bacterial component of the canola rhizosphere microbiota cannot only be stable across pedoclimatic zones but also through years. This core bacterial component was formed of only one taxon, ASV1 identified as cf. *Pseudarthrobacter* sp., which was also identified as a hub taxon and had a cohort of seven bacterial taxa with stable relationships across the two years of the study.

### ASV1, cf. *Pseudarthrobacter* sp.

ASV1 was the only bacterial member that fit the definition of a core microbiota member that was detected in the canola rhizosphere and it was the most abundant ASV in both years of sampling. With our current sequencing technology (Illumina MIseq), it is likely that prokaryotes can go unseen if their abundance is low in a sample. ASV1 was the only bacterial core member identified, but it is probable that other less abundant prokaryotic members of this core microbiota were undetected. Furthermore, 16S rRNA gene sequences obtained with Illumina MiSeq technology do not have enough taxonomic resolution to distinguish between closely related species and uncertainty exists: ASV1 matches with 100% identity with at least 100 *Arthrobacter* and *Pseudarthrobacter* sequences in NCBI database. *Arthrobacter* is a genus of gram-positive bacteria from the *Micrococcaceae* family that was subdivided in several other genera like *Pseudarthrobacter* (Busse, 2016). This genus includes mainly soil bacterial species (Busse, 2016). *Arthrobacter* is also a genus with many species known as PGPB (Chan and Katznelson, 1961; Manzanera et al., 2015; Ullah and Bano, 2015; Aviles-Garcia et al., 2016; Fincheira and Quiroz, 2018) colonizing the roots and rhizosphere of a large spectrum of agricultural crops, such as rice or tomato. Lay et al. (2018) reported a member of canola rhizosphere core microbiota identified as *Arthrobacter* that shared 100% identity with ASV1 in similar sites of the Canadian Prairies in 2014. They also reported that their *Arthrobacter* was positively correlated with canola yield as it was the case here with ASV1 in 2016. Furthermore, an *Arthrobacter* sp. was previously shown to increase canola yield and acts as PGPB (Kloepper, 1988). This genus was reported as a highly competitive and fast growing bacteria in canola rhizosphere (Tkacz et al., 2015). Lay et al. (2018) also reported the presence of *Arthrobacter* sp. in wheat and pea rhizospheres in rotation with canola, but in smaller proportions than in canola rhizosphere. That omnipresence and abundance of ASV1 (cf. *Pseudarthrobacter* sp.) in all our plots suggest a selection by canola and highlight this taxon as a good PGPB candidate.

### Variations in Bacterial Microbiota

Bacterial communities are known to be sensitive to changes in abiotic factors such as pH and humidity, or nutrient availability (Norman and Barrett, 2016; Wan et al., 2020). As plants actively control their rhizosphere microbiota through root exudates (Bais et al., 2006; Eisenhauer et al., 2017), we expected important differences in the bacterial communities of our crop diversification treatments. This was not the case. In 2013, no effect of crop rotation on bacterial community structure was detected and in 2016, the only significant difference was between the two extreme treatments, i.e., canola monoculture and the highest level of crop diversification, and the difference was marginally significant (*P* = 0.047). Indicator species analysis showed those two crop diversification treatments as the ones that had the highest number of indicator species. It is possible that the number of indicator species (26) of the monoculture in 2016 with a dominance of *Chloroflexi* (Table 4) could be the source of the difference in community structure, with the highest level of crop diversification with the BMRPP, even if no significant differences was found in 2013 between those two crop diversification treatments. Long lasting effect of agricultural management such as crop rotation were reported in the literature (Buckley and Schmidt, 2001). In the Brazilian Amazon for example, crop management seems to have a significant impact on microbial community structure (Jesus et al., 2009). For temperate environments, our results are consistent with Jesus et al. (2016) who did not find any influence of crop rotation on soil microbial communities in Michigan.

In our study, we examined the bacterial community in the canola rhizosphere, a component of the microbiota that is principally influenced by canola root exudates (Rumberger and Marschner, 2003), mitigating the effects of other crops in the rotation systems. We do not know if the crop diversification levels influenced the bulk soil bacterial communities. However, our results showed that canola recruited similar bacterial communities between all crop diversification levels in 2013. Even if most of the microbes in the rhizosphere are probably selected by the plant from its surrounding soil, it is also possible that a part of the canola rhizosphere microbiota can be inherited maternally with the seed microbiome as it is known to be the case for a wide range of plants (Shade et al., 2017). That could explain the similarities of canola rhizosphere community structure in systems with different levels of diversification. It is also possible that the bacterial communities in our diversified system were not host-specific, but colonize the roots of all crop species used in rotation, as it was reported by Lay et al. (2018). They found that the bacterial microbiota of canola rhizosphere was more similar to the one found in pea than the one found in wheat rhizosphere. But here, we did not find significant difference in community structure between the low, medium and high crop diversification in 2013 and only a slightly significant difference in 2016, suggesting that rotation crops have a limited influence on the bacterial communities of canola rhizosphere. Thus, we can consider the influence of abiotic variation on bacterial community in our study. A previous study showed that soil type and the frequency of rainfall have stronger effects on the microbial community of canola rhizosphere than crop rotations (Schlatter et al., 2019). Floc’h et al. (2020) also found a large variation in fungal rhizosphere community structure that was linked with difference in water availability in canola rhizosphere. In the present study, the experimental plots and sampling times were the same as those used in Floc’h et al. (2020). But the difference in precipitation between years (Supplementary Figure S2) did not affect the stability of the bacterial community structure observed in 2013 and 2016, contrarily to what was found for the fungal community in Floc’h et al. (2020). This stability is noteworthy. Bacterial interactions in canola rhizosphere microbiota also showed stability through years, here.

### Interactions in the Bacterial Component of the Microbiota

Using the same rhizosphere soil samples, Floc’h et al. (2020) reported drastic changes between years in the dynamics of fungal interactions in the microbiota of canola rhizosphere. In the present work, if the complexity of the interaction network changed between the two years of sampling, the pool of bacteria forming its nucleus remained the same. The hotspot of interaction was always articulated around ASV1 (*Pseudarthrobacter* sp.). ASV1 was the only core bacterial member of the microbiota of canola rhizosphere and the only hub taxa detected with network analysis for both years of the present study. The fungal hub taxa in canola rhizosphere were subject to change between the years of the study, but it was not the case for bacterial hub taxa.

For both year of sampling, ASV1 was interacting with seven other taxa: ASV2 (cf. *Yersinia* sp.), ASV3 (cf. *Nitrososphaeraceae* sp.), ASV4 (cf. *Stenotrophomonas* sp.), ASV6 (cf. *Chloroflexi* KD4-96), ASV11 (cf. *Stenotrophomonas* sp.), ASV25 (cf. *Candidatus Nitrosocosmicus* sp.) and ASV71 (cf. *Paenarthrobacter* sp). The persistence of these interactions across time suggests a close interaction of ASV1 with these other members of the community. The fact that ASV6 was negatively linked with ASV1 and negatively correlated with canola yield raises interest. This phylum is associated with several agricultural plants like potato (Ýnceoğlu et al., 2011), lettuce (Cardinale et al., 2015) or maize (Peiffer et al., 2013) and was found in a large spectrum of soil ecosystems including forest, grassland, and tundra ecosystems (Fierer et al., 2012). *Chloroflexi* appears as characteristic of the rhizosphere of canola monoculture: 3 of 9 ASVs in 2013 and 9 of 26 ASVs were identified as indicator species in 2016 (Table 4). Monoculture of canola was found to have lower yield values across time and favour accumulation of microbial pathogenic taxa in soil (Hummel et al., 2009; Harker et al., 2015). *Chloroflexi* have been reported in the canola rhizosphere previously, but there was no mention of *Chloroflexi* species being pathogenic to canola (Gkarmiri et al., 2017). Correlations do no indicate that there is a causal relationship between the abundance of the different bacterial ASVs and canola yield. Correlations may point to bacteria that benefit from higher canola growth, or to a condition favorable to both canola and these bacteria, rather than an effect of the bacteria on plant productivity. However, the correlation values can be used as an index for identifying potential bacterial ASV of interest for the enhancement of canola production, since the bacteria directly beneficial to canola would be among those showing positive correlation with yield. It is possible that ASV6 could be commensal of canola fungal pathogens or of other microbes that are favored by monoculture (Floc’h et al., 2020), or pathogenic itself. Tests of pathogenicity should be made, or cross-kingdom network interactions studies conducted to verify the occurrence of ASV6 with pathogenic microbes.

In the cohort of taxa associated with ASV1, two other taxa were positively correlated with canola yield in 2016: ASV3 and ASV71. ASV71 was identified as *Arthrobacter*, so it is phylogenetically closely related to ASV1, and could be a potential PGPB with ASV1 (Manzanera et al., 2015; Ullah and Bano, 2015; Pereira et al., 2019). ASV3 is an archaea identified as a member of the *Nitrososphaeraceae* family that was poorly correlated with canola yield. Little information about this family is available. The presence of *Nitrososphaeraceae* was previously reported by Gkarmiri et al. (2017), and Lay et al. (2018) found core microbiota members of canola rhizosphere that were genetically close to *Nitrocosmicus* spp. Another study mentioned *Nitrososphaeraceae* as a microbial taxa retrieved from spacecraft surfaces (La Duc et al., 2012). This family appears to be widely distributed in the environment. As hub taxa can have very strong influence on the whole microbiota and on plant performance, ASV1 and its cohort members could be important. These bacteria should be isolated and tested under controlled conditions in structured experiments to examine their potential PGPB activity or pathogenic behavior on canola.

## Conclusion

In this work, we have shown that the bacterial component of the core microbiota of canola rhizosphere is stable across years despite dissimilarity in precipitations. We identified the single core bacterial ASV in the microbiota of canola rhizosphere as cf. *Pseudarthrobacter* sp. In both years of the study, this single bacterial core microbiota member was a hub taxon in stable association with a cohort of bacteria. *Chloroflexi* were somewhat typical of canola monoculture, but the influence of crop diversification level on bacterial community structure, was only marginal, showing that the bacterial component of the microbiota of canola rhizosphere is more stable than its fungal component. This study provides information about bacterial and archaeal species in canola rhizosphere that could be important for future enhancement of canola production through microbiota manipulation or development of new cohorts for bio-inoculants.

## Data Availability Statement

The MiSeq sequencing data generated as part of this work are publicly available on Zenodo (https://zenodo.org/record/3626047#.XisHASZOmV4).

## Author Contributions

J-BF and CH designed and performed the experiment. MS-A, CH, and MH supervised the project. NL, KH, CH, and MS-A provided the material and analytic tools. J-BF analyzed the data. J-BF, MS-A, and CH wrote the manuscript. All authors revised and approved the manuscript.

## Conflict of Interest

The authors declare that the research was conducted in the absence of any commercial or financial relationships that could be construed as a potential conflict of interest.

## Footnotes

1 http://www.earthmicrobiome.org/emp-standard-protocols/16s/

2 https://zenodo.org/record/3626047#.XisHASZOmV4

## Supplementary Material

FIGURE S1 Rarefaction curves for each rhizosphere soil sample, showing the relationship between the number of ASVs and the abundance of 16S rRNA gene sequences reads, in the 2013 and 2016 dataset.

FIGURE S2 Cumulative precipitation proportion at each site in 2013 and 2016.

FIGURE S3 PCOA analysis of prokaryotic communities of canola rhizosphere using Hellinger distances in 2013.

FIGURE S4 PCOA analysis of prokaryotic communities of canola rhizosphere using Hellinger distances in 2016.
