# Supplementary figures and images for: Bacterial Communities of the Canola Rhizosphere: Network Analysis Reveals a Core Bacterium Shaping Microbial Interactions
Source: Front Microbiol. 2020 Jul 10;11:1587. doi: 10.3389/fmicb.2020.01587 (PMC7418181; doi:10.3389/fmicb.2020.01587)

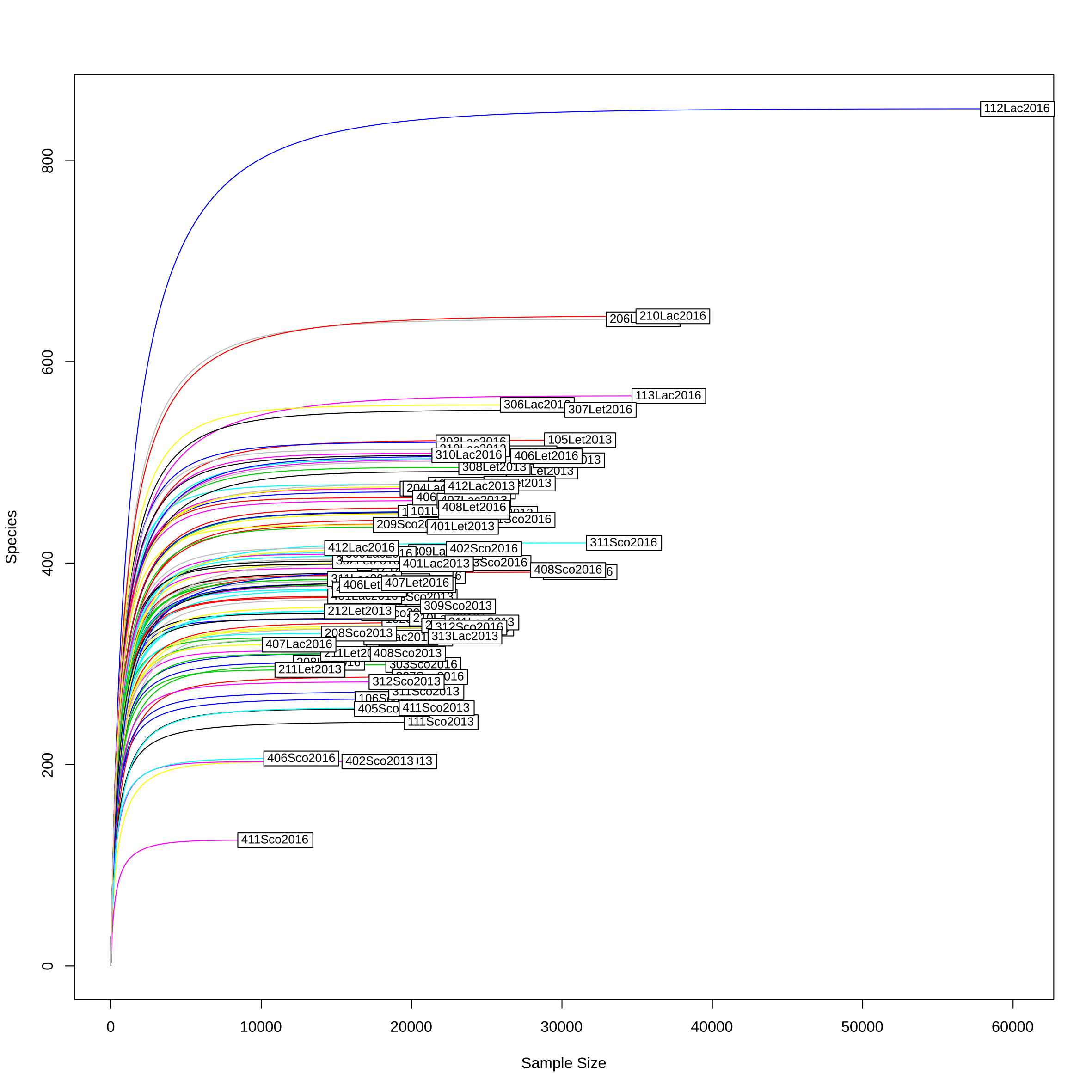

Supplement: FIGURE S1 — Rarefaction curves for each rhizosphere soil sample, showing the relationship between the number of ASVs and the abundance of 16S rRNA gene sequences reads, in the 2013 and 2016 dataset. [file Image_1.TIF]

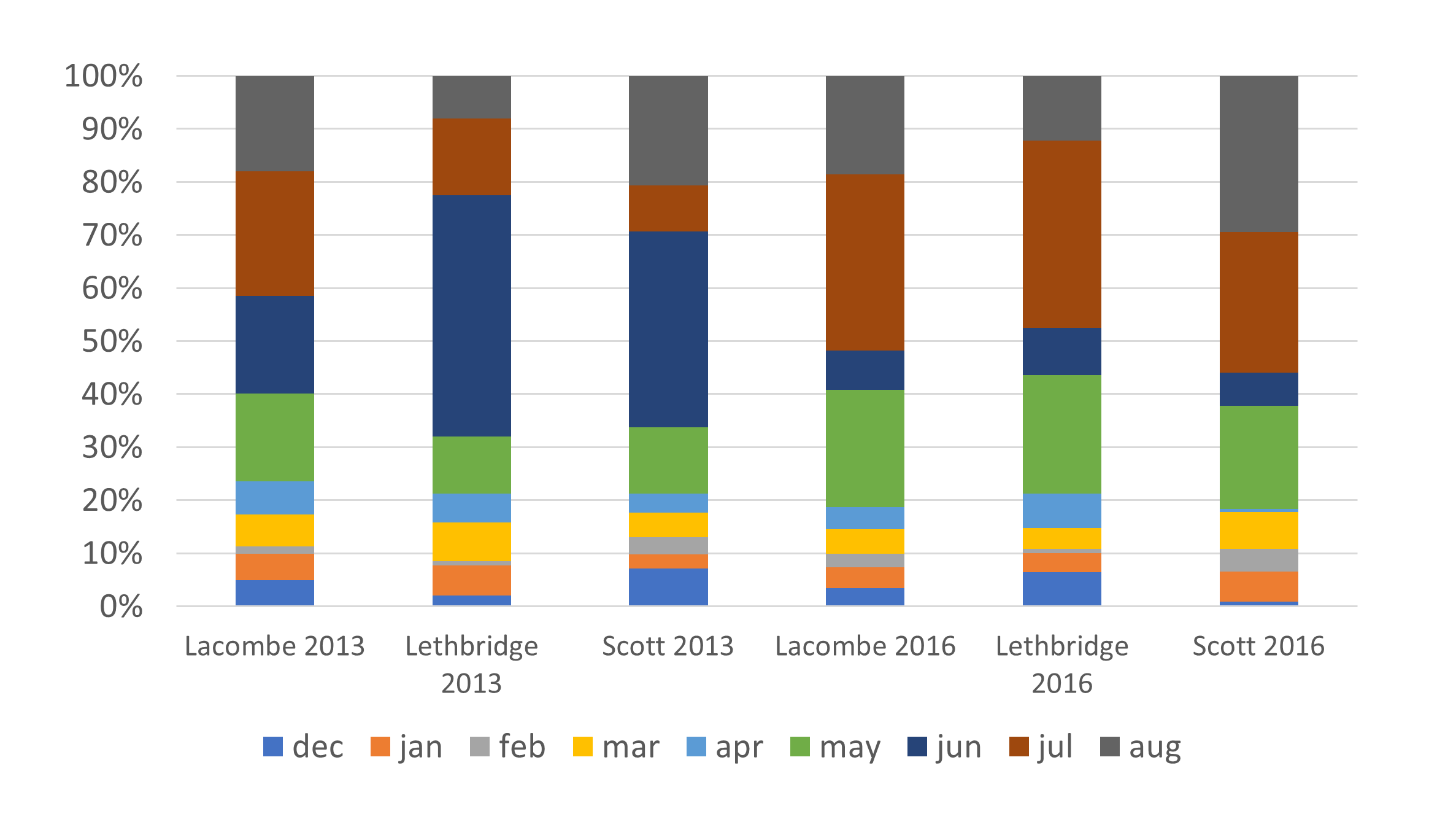

Supplement: FIGURE S2 — Cumulative precipitation proportion at each site in 2013 and 2016. [file Image_2.TIF]

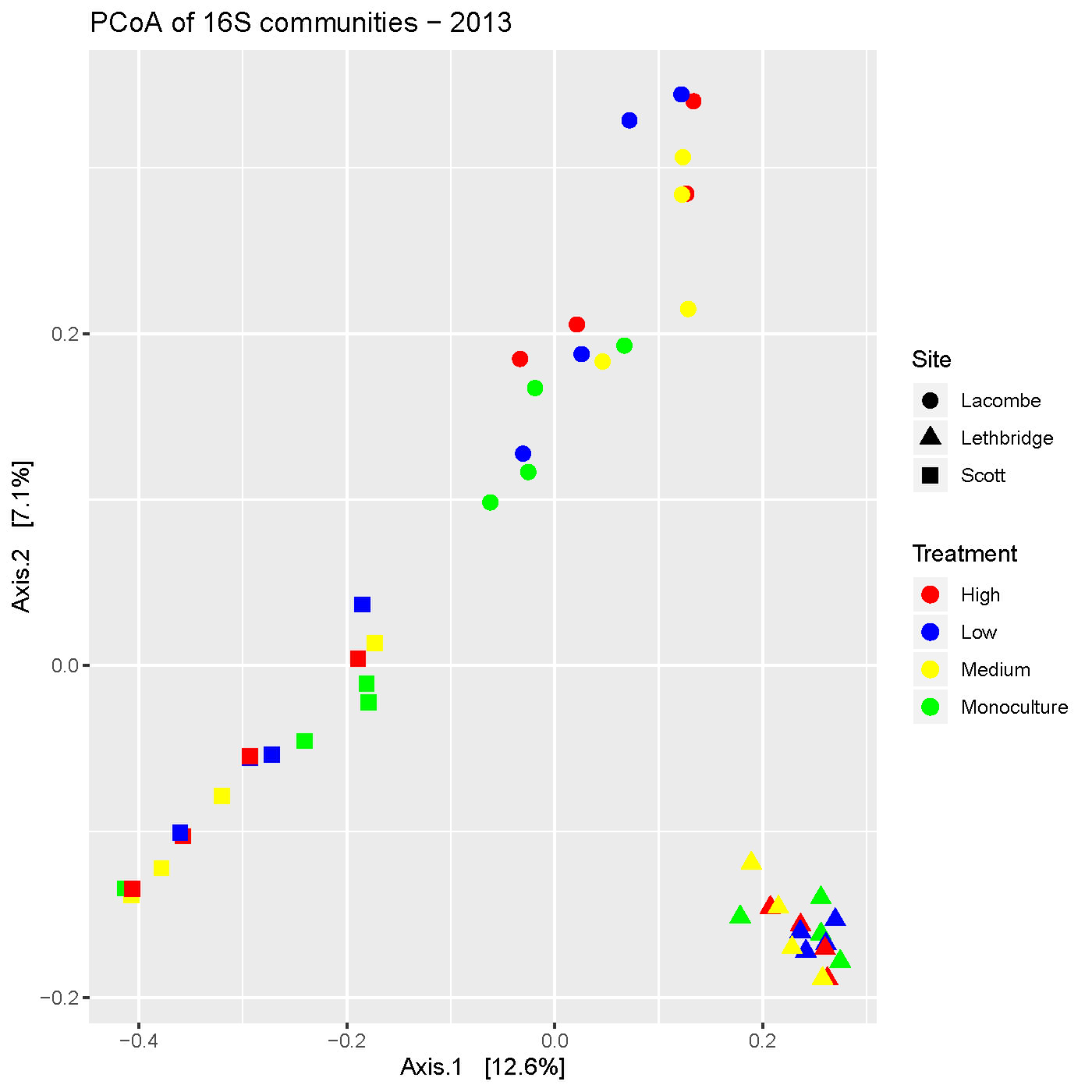

Supplement: FIGURE S3 — PCOA analysis of prokaryotic communities of canola rhizosphere using Hellinger distances in 2013. [file Image_3.TIF]

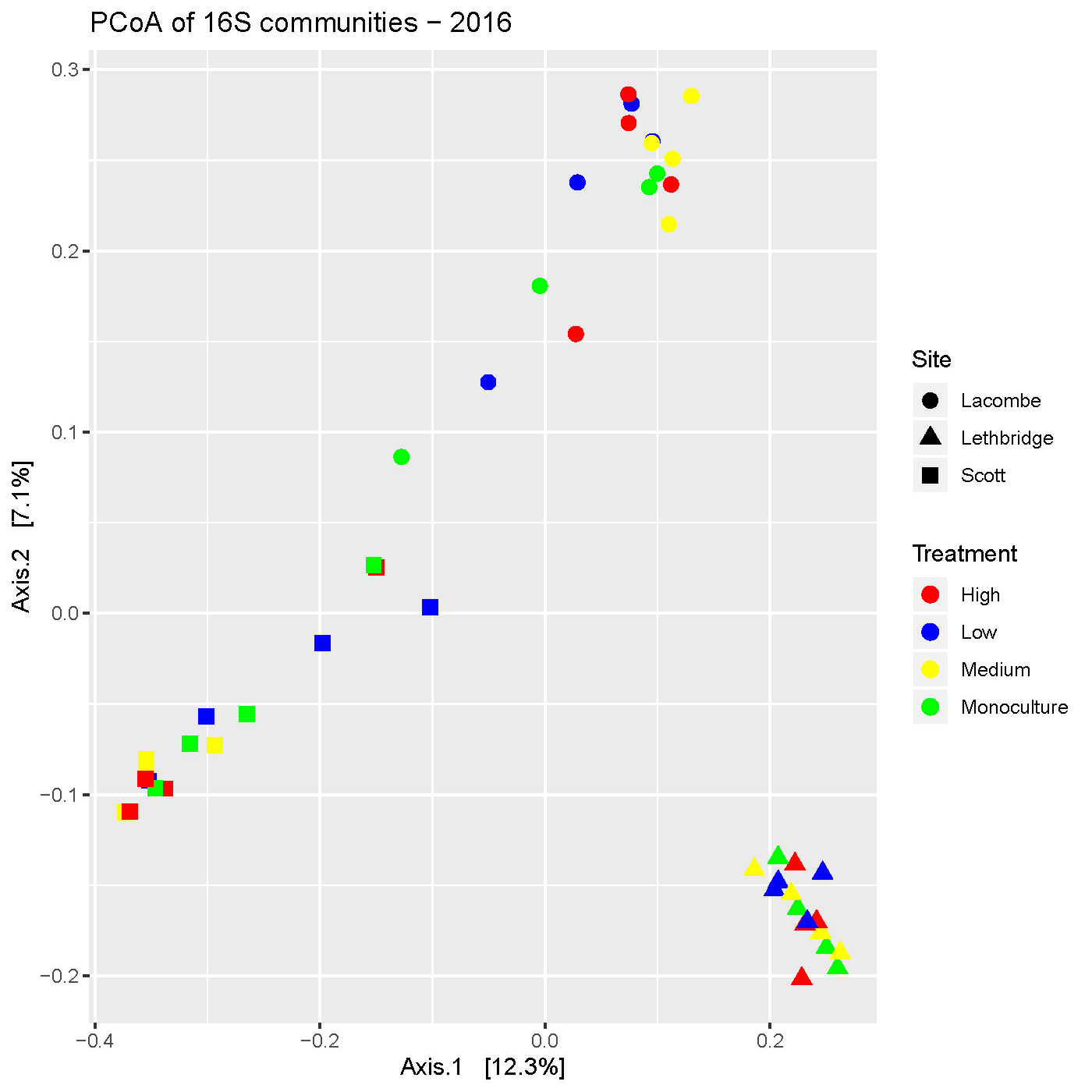

Supplement: FIGURE S4 — PCOA analysis of prokaryotic communities of canola rhizosphere using Hellinger distances in 2016. [file Image_4.TIF]
